# IP_3_R-mediated Ca^2+^ release regulates protein metabolism in *Drosophila* neuroendocrine cells: implications for development under nutrient stress

**DOI:** 10.1242/dev.145235

**Published:** 2017-04-15

**Authors:** Megha, Gaiti Hasan

**Affiliations:** National Centre for Biological Sciences, Tata Institute of Fundamental Research, Bangalore 560065, India

**Keywords:** Insulin signaling, ER Ca^2+^ stores, dILP5, Starvation, Pupariation

## Abstract

Successful completion of animal development is fundamentally reliant on nutritional cues. Surviving periods of nutritional insufficiency requires adaptations that are coordinated, in part, by neural circuits. As neuropeptides secreted by neuroendocrine (NE) cells modulate neural circuits, we investigated NE cell function during development under nutrient stress. Starved *Drosophila* larvae exhibited reduced pupariation if either insulin signaling or IP_3_/Ca^2+^ signaling were downregulated in NE cells. Moreover, an IP_3_R (inositol 1,4,5-trisphosphate receptor) loss-of-function mutant displayed reduced protein synthesis, which was rescued by overexpression of either InR (insulin receptor) or IP_3_R in NE cells of the mutant, suggesting that the two signaling pathways might be functionally compensatory. Furthermore, cultured IP_3_R mutant NE cells, but not neurons, exhibited reduced protein translation. Thus cell-specific regulation of protein synthesis by IP_3_R in NE cells influences protein metabolism. We propose that this regulation helps developing animals survive in poor nutritional conditions.

## Abstract (precis)

**Summary:** Intracellular Ca^2+^ signaling regulates protein translation and can compensate for insulin signaling in specialized neuro-hormonal cells, thus enabling *Drosophila* larval to pupal development under acute starvation.

## INTRODUCTION

Nutritional poverty during development has long-lasting effects on the growth and behavior of an animal. Although under-nutrition causes overall body size to decrease, the brain grows to near-normal size, a process termed ‘brain sparing’ (Dobbing and Sands, 1971). This suggests unique mechanisms in neuronal tissues to weather nutritional stress. *Drosophila* is an attractive model system to uncover these mechanisms because larvae subjected to nutrient restriction exhibit ‘brain sparing’ (Cheng et al., 2011) and nutritional effects on larval-to-pupal development are easily monitored. Additionally, growth signaling pathways activated by dietary cues such as insulin receptor (InR) and TOR signaling, are conserved in *Drosophila* (Padmanabha and Baker, 2014).

When starved, larval neural stem cells (NSCs) continue to proliferate by using an InR ortholog, Alk (Anaplastic lymphoma kinase) (Cheng et al., 2011). This study focuses on neuroendocrine (NE) cells, which, unlike NSCs, are differentiated and non-dividing. Importantly, neuropeptides released by NE cells modulate neural circuits that regulate processes associated with animal physiology and behavior (Nässel and Winther, 2010; Taghert and Nitabach, 2012), eventually influencing how animals adapt to external or internal stimuli. Crucially, NE cells produce peptide hormones that regulate feeding behavior and metabolism (Nässel and Winther, 2010), processes required for larvae to complete development successfully.

IP_3_R (Itp-r83A in *Drosophila*) is an endoplasmic reticulum (ER) channel that releases stored Ca^2+^ and acts downstream of G protein-coupled receptor activation. The ER-resident protein STIM (Stromal interaction molecule) conveys loss of stored Ca^2+^ to Orai (Olf186-F in *Drosophila*), a plasma membrane Ca^2+^ channel, thereby enabling store-operated Ca^2+^ entry (SOCE) from the extracellular milieu. SOCE occurs in both mammals (Prakriya and Lewis, 2015) and flies (Agrawal et al., 2010; Venkiteswaran and Hasan, 2009). Thus, all three molecules – IP_3_R, STIM and Orai – function during stimulus-dependent elevation of cytosolic Ca^2+^ that potentiates diverse signaling outcomes, depending on the cellular context.

Loss of IP_3_R (Subramanian et al., 2013b) and STIM (Baumbach et al., 2014) leads to obesity in adult *Drosophila*. Importantly, adults of a hypomorphic IP_3_R mutant heteroallelic combination, *itpr^ka1091/ug3^* (hereafter: *itpr^ku^*) exhibit obesity, starvation resistance and hyperphagia, which are all rescued by overexpression of IP_3_R in NE cells (Subramanian et al., 2013a). This adult metabolic phenotype prompted us to investigate the role of IP_3_R and InR in NE cells during larval development.

## RESULTS AND DISCUSSION

### Downregulation of InR- or IP_3_R-mediated intracellular Ca^2+^ signaling in NE cells reduces pupariation under starvation

**Fig. 1.**
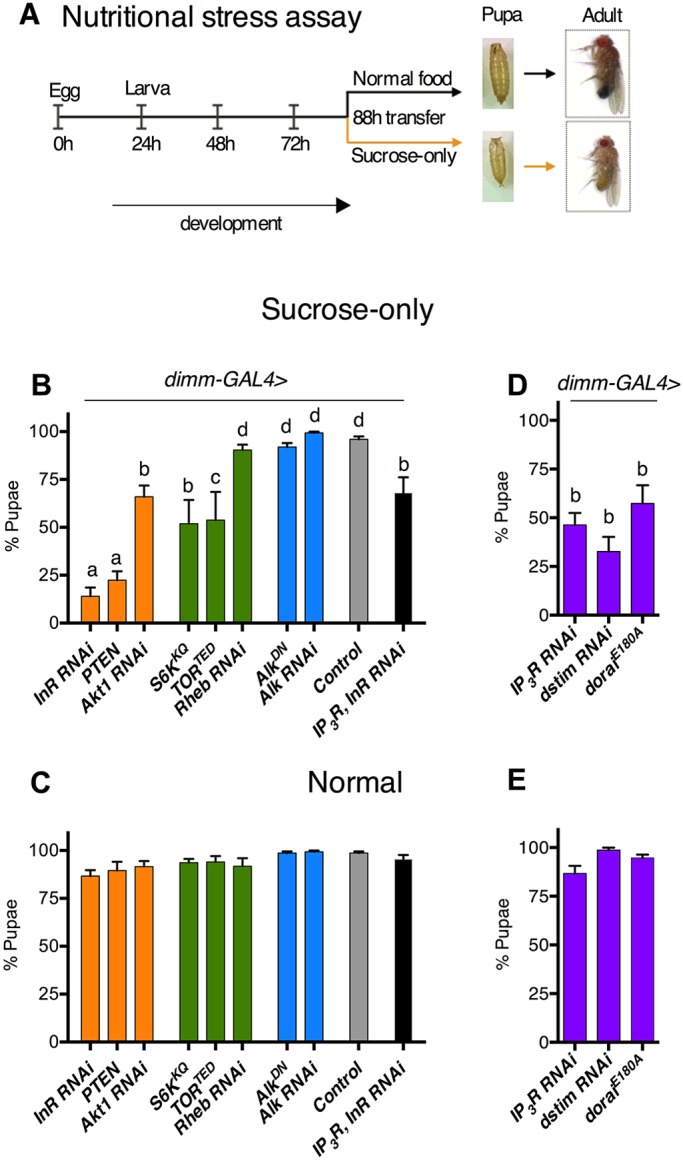
**Downregulation of InR, TOR and intracellular Ca^2+^ signaling pathways in NE cells impairs larval development on sucrose.** (A) Schematic of assay protocol with representative examples of pupa and adult appearance. (B,C) Pupariation upon reduction of InR/TOR signaling in *dimm-GAL4* cells on sucrose (B) or normal diet (C). (D,E) Pupariation upon reduction of intracellular Ca^2+^ signaling on sucrose (D) or normal diet (E). Regulators of the InR (orange), TOR (green), Alk (blue) or intracellular Ca^2+^ (purple) signaling. UAS controls are shown in Fig. S1. Bars with the same letter represent statistically indistinguishable groups (one-way ANOVA with post-hoc Tukey's test, *P*<0.05). *n*=6 batches of 25 larvae each. Data represent mean±s.e.m.

In *Drosophila*, a large subset of NE cells express the transcription factor DIMM (Park et al., 2008). We downregulated InR, TOR and intracellular Ca^2+^ signaling pathways in *dimm^+^* NE cells using the UAS-GAL4 system (Brand and Perrimon, 1993), and monitored pupariation of larvae on a sucrose-only diet from 88 h after egg laying (AEL; Fig. 1A), a time point used previously (Cheng et al., 2011).

Less than 25% of larvae pupariated on sucrose either after *InR* knockdown or after overexpression of a negative regulator of InR signaling, *Pten* (Fig. 1B). Manipulation of InR/TOR signaling components by overexpression of dominant-negative versions (*TOR^TED^*, *S6K^KQ^*) or RNAi (*Akt1*, *Rheb*) affected pupariation mildly or not at all (Fig. 1C). Unlike NSCs (Cheng et al., 2011), neither overexpression of dominant-negative *Alk* (*Alk^DN^*) nor reduction via *Alk* RNAi, in NE cells, affected larval development, regardless of diet (Fig. 1B,C). Perhaps because NE cells are differentiated, they employ another mechanism to maintain insulin signaling during starvation. InR/TOR signaling affects NE cell size (Luo et al., 2013) and, interestingly, the pupariation rate that we observed in larvae on a sucrose-only diet correlates with the observations of Luo et al. For example, *InR* knockdown resulted in a NE cell size reduction of ∼18% (Luo et al., 2013) and gave a strong phenotype in our assay, whereas reduction of *Rheb* or *Alk*, which does not change NE cell size, gave no phenotype in our assay (Fig. 1B). Robust pupariation on normal food for the above genetic manipulations (Fig. 1C) suggested that dietary nutrients compensate for reduced InR/TOR signaling in NE cells. Together, these observations underscore the importance of NE cell function in overcoming nutrient stress.

Reducing intracellular Ca^2+^ signaling in NE cells by knockdown of either IP_3_R or dSTIM, or overexpression of a dominant-negative form of Orai (*Orai^E180A^*) (Pathak et al., 2015), reduced pupariation on the sucrose-only diet (Fig. 1D) but not on normal food (Fig. 1E). The similarities in outcome upon downregulation of either InR/TOR signaling or intracellular Ca^2+^ signaling prompted us to test the genetic interactions among components of the two pathways. Overexpression of IP_3_R in NE cells with InR knockdown led to increased pupariation on sucrose, compared with InR reduction alone (Fig. 1B), suggesting that under nutrient stress, IP_3_R can compensate for InR. Next, we investigated the IP_3_R mutant, *itpr^ku^*.

### IP_3_R mutant larvae are deficient in protein metabolism

**Fig. 2.**
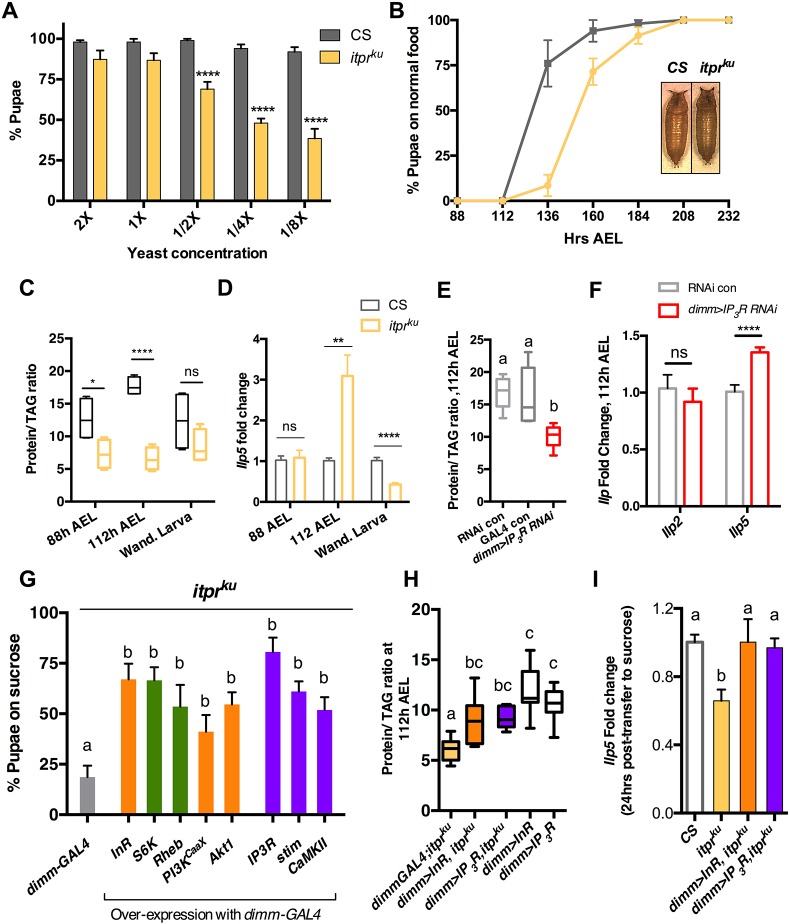
**Dysregulated protein metabolism in the IP_3_R hypomorph *itpr^ku^* can be rescued by overexpression of either InR or IP_3_R in NE cells*.*** (A) Pupariation of 65 h larvae transferred into media with varying amounts of yeast. Two-way ANOVA, ****P<0.0001. (B) Pupariation over time after transfer to normal food at 88 h. Inset shows representative pupa. Relative pupal volume is shown in Fig. S2C. (C,E) Temporal changes in protein/TAG ratio, normalized to weight, for different genotypes. *n*≥5. See also Fig. S2D-F. (D,F) *Ilp5* transcript levels in larval CNS normalized to *rp49*. *n*=6. (G) Pupariation of *itpr^ku^* on sucrose diet upon overexpression of positive regulators of InR and TOR signaling (orange and green) or intracellular Ca^2+^ signaling (purple) in NE cells. See also Fig. S3D. (H) Protein/TAG ratios normalized to weight. See also Fig. S2D-F. *n*≥8. (I) *Ilp5* transcript levels in larval CNS normalized to *rp49*. *n*=4. Statistics: C,D,F, unpaired *t*-test, *P<0.05, **P<0.01, ****P<0.0001; E,G,H,I, one-way ANOVA. Bars with the same letter represent statistically indistinguishable groups (one-way ANOVA with a post-hoc Tukey's test, *P*<0.05). Data represent mean±s.e.m. CS, *Canton S*; ns, not significant; Wand., wandering.

Although *itpr^ku^* exhibited robust pupariation on normal food (Fig. S2A), its pupariation was sensitive to reduction of yeast, the major source of dietary protein in ‘normal food’ (Fig. 2A). Pupariation was also reduced on sucrose (Fig. S2A), and rescued by supplementation with amino acids (Jayakumar et al., 2016) or amino acids and vitamins, but not lipids or vitamins alone (Fig. S2A).

At 88 h, an equal proportion of second (2L) and third (3L) instar *itpr^ku^* larvae co-exist (Fig. S2B), suggesting pleiotropic development delay. Additionally, when the pupariation rate of 88 h 3Ls was monitored, *itpr^ku^* displayed a lag of ∼24 h (Fig. 2B). Surprisingly, longer development time did not result in greater pupal volume (Fig. 2B; Fig. S2C), typically seen when larvae spend more time feeding (McBrayer et al., 2007). Although the weight of 3L *itpr^ku^* at 88 h, 112 h and as wandering larvae were not different from control (Fig. S2D), protein and triacylglyceride (TAG) levels were different (Fig. S2E,F). At 88 h and 112 h, *itpr^ku^* had higher TAG levels and lower protein levels. In wandering 3L, these levels were near normal (Fig. S2E,F). When plotted as protein/TAG ratio (Fig. 2C), it appeared that *itpr^ku^* had a slower rate of protein assimilation. Increased developmental time on normal food by *itpr^ku^* might therefore be a strategy to accumulate sufficient protein, and also explain why it does not result in increased body size.

Abnormal protein/TAG ratios suggested perturbed insulin signaling in *itpr^ku^*. We therefore measured transcript levels of *Drosophila* insulin-like peptides (dILPs) 2, 3, 5 and 6 from larval brains on normal food (Fig. 2D; Fig. S3A). Except *Ilp5*, which varied temporally to a significant degree (Fig. 2D), the trend for other dILPs (Fig. S2F) was similar to control. Although produced in the same set of NE cells (insulin-producing cells, IPCs), *Ilp2*, *Ilp3* and *Ilp5* transcripts are independently regulated. *Ilp2* transcription is considered to be a systemic response, whereas *Ilp3* is regulated by sugar (Kim and Neufeld, 2015), and *Ilp5* by protein concentration (Geminard et al., 2009; Okamoto and Nishimura, 2015). Selective variation of *Ilp5* thus indicated dysfunctional protein sensing in *itpr^ku^*. Overexpression of *Ilp2* results in larger adults (Sato-Miyata et al., 2014); in contrast, the size of *itpr^ku^* pupae (Fig. S2C) is similar to that of controls, suggesting that the small increase of *Ilp2* at 112 h (Fig. S2F) could be a response to *Ilp5* upregulation.

We next downregulated IP_3_R in various cells/organs known to coordinate metabolism and development (Fig. S3B). As expected, IP_3_R knockdown in the prothoracic gland (PG) decreased pupariation on sucrose (Fig. S3B) because IP_3_R is required for ecdysone release from the PG (Venkatesh and Hasan, 1997; Yamanaka et al., 2015). However, unlike *itpr^ku^* or larvae with reduced IP_3_R in either NE cells or all neurons (Fig. S3B), supplementation with amino acids did not improve viability. This suggested that IP_3_R functions differently in PG cells and neurons. It is also likely that PG function in *itpr^ku^* is not as compromised as it is in the PG-IP_3_R-knockdown condition, as Ca^2+^ release in *itpr^ku^* neurons is reduced but not abolished (Joshi et al., 2004; Srikanth et al., 2004; Venkiteswaran and Hasan, 2009). Notably, reduction of IP_3_R in the fat body, or oenocytes (Fig. S3B), other sites of metabolic regulation in *Drosophila*, had no effect on larval development on sucrose.

IP_3_R reduction in NE cells also resulted in larvae with a lower protein/TAG ratio (Fig. 2E) and elevated *Ilp5* expression (Fig. 2F). These features were similar (although reduced in magnitude) to *itpr^ku^* (Fig. 2C,D), suggesting the contribution of non-NE cells to *itpr^ku^* phenotype. Indeed, a set of glutamatergic neurons have been identified in which overexpression of IP_3_R is sufficient to rescue lethality of *itpr^ku^* on sucrose (Jayakumar et al., 2016).

Because *itpr^ku^* displayed abnormal transcript levels of *Ilp5* and *Ilp2*, loss of IP_3_R specifically in the IPCs was tested, and was found not to affect pupariation on sucrose (Fig. S3B)*.* This is consistent with previous observations that IP_3_R knockdown in IPCs does not phenocopy IP_3_R mutant phenotypes (Agrawal et al., 2009; Subramanian et al., 2013a). Together, these data suggest that IP_3_R in the IPCs does not affect dILPs directly. Increases in *Ilp5* and *Ilp2* transcripts in *itpr^ku^* (Fig. 2D; Fig. S3A) might instead be diet-dependent compensatory systemic responses. This is supported by the modest rescue of *itpr^ku^* pupariation on sucrose, with overexpression of either *Ilp2* in IPCs (Jayakumar et al., 2016) or *Ilp5* in NE cells (Fig. S3C).

### Increasing InR/TOR or intracellular Ca^2+^ signaling in NE cells restores protein synthesis levels in the IP_3_R mutant

Next, we tested the effect of upregulation of InR and intracellular Ca^2+^ signaling components in NE cells of *itpr^ku^*. Overexpression of positive regulators of either the InR (*InR*, *PI3K^CaaX^*, *Akt1*) or the TOR (*S6K*, *Rheb*) pathway rescued *itpr^ku^* development under nutritional stress (Fig. 2G; Fig. S3D). These manipulations increase growth by promoting ribosomal biogenesis (Grewal, 2009), and in NE cells by increasing their size (Luo et al., 2013). Restoring intracellular Ca^2+^ signaling by overexpression of wild-type IP_3_R or STIM, as well as overexpression of *CaMKII*, a kinase that propagates Ca^2+^ signaling, in NE cells of *itpr^ku^*, also rescued larval lethality on sucrose (Fig. 2G; Fig. S3D).

At the systemic level, overexpression of either InR or IP_3_R in NE cells (*dimm>InR/IP_3_R*, *itpr^ku^*) was sufficient to increase protein/TAG ratios of *itpr^ku^* (Fig. 2H) at 112 h to levels similar to wandering stage *itpr^ku^* on normal food (Fig. 2C), suggesting that both pathways ultimately affected systemic protein metabolism. Protein/TAG ratios of rescues (*dimm>InR/IP_3_R, itpr^ku^*) are compared with *dimm>InR/IP_3_R* because non-linear increases in weight, protein and TAG levels were observed when either InR or IP_3_R alone were overexpressed in NE cells (Fig. S2D-F). Of note are TAG levels in *dimmGAL4*;*itpr^ku^* control and *dimm>InR/IP_3_R itpr^ku^* rescues (Fig. S2E). In both rescue conditions, protein levels increase (Fig. S2F), whereas TAG levels remain high (like *dimmGAL4*;*itpr^ku^*). Thus, insufficient protein, and not higher TAGs, correlates with the pupariation defect of *itpr^ku^* on sucrose.

As overexpression of *Ilp5* rescued *itpr^ku^* partially and *itpr^ku^* displayed upregulated *Ilp5* at 112 h on normal food, we investigated whether *dimm>InR/IP_3_R* rescues involved *Ilp5*. Nutrient withdrawal typically reduces *Ilp2*, *Ilp3* and *Ilp5* transcript levels significantly (Ikeya et al., 2002) and 88 h control larvae tested for levels of these dILPs after 24 h on sucrose showed expected reductions (Fig. S3E). Interestingly, *itpr^ku^* displayed greater reduction in *Ilp5* (Fig. 2I) but not *Ilp2* (Fig. S3F), when tested 24 h after transfer to sucrose. This reduction in *Ilp5* probably affects *itpr^ku^* because at this time point (112 h) on normal food, it requires a ∼3-fold upregulation of *Ilp5* (Fig. 2D). On sucrose, overexpression of InR or IP_3_R in NE cells increases *Ilp5* levels in *itpr^ku^* (Fig. 2I) to control levels, without affecting *Ilp2* levels (Fig. S3F). This suggests that *dimm>InR/IP_3_R* rescues *itpr^ku^* in part by systemically upregulating *Ilp5*.

### IP_3_R positively regulates protein translation in NE cells

**Fig. 3.**
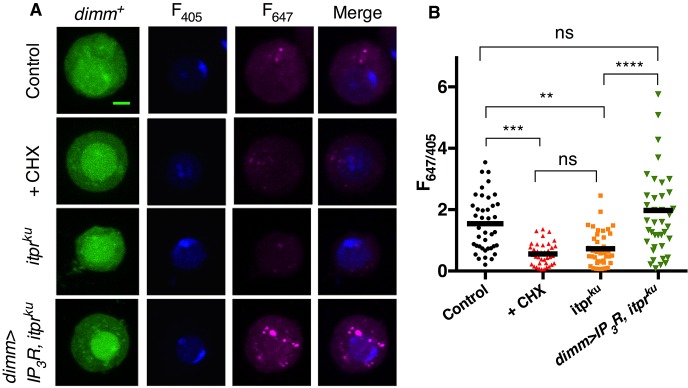
**NE cells from *itpr^ku^* display reduced protein synthesis.** (A) Representative confocal images of NE cells (*dimm^+^*, GFP positive) in culture from the indicated genotypes and conditions. Newly synthesized peptides (*F*_647_) and nuclear volume (*F*_405_) were measured. Cells were treated with 10 µM cycloheximide (CHX) for 30 min. Scale bar: 2 µm. (B) Quantification of *F*_647_ and *F*_405_ from confocal images. *n*≥40. One-way ANOVA with post-hoc Holms–Sidak. **P<0.01, *** *P*<0.001, ****P<0.0001. Data represent mean±s.e.m. ns, not significant.

As systemic protein levels in *itpr^ku^* were rescued by overexpression of IP_3_R in NE cells, a cellular role for IP_3_R in protein translation was investigated. In *itpr^ku^* NE cells, obtained by culturing larval CNS, protein translation was reduced by ∼50%, similar to NE cells treated with the protein synthesis inhibitor cycloheximide (Fig. 3A,B). This reduction was rescued by overexpression of IP_3_R (Fig. 3A,B), strengthening the idea that IP_3_R, like InR, has a positive effect on protein synthesis.

This observation is opposite to that reported for mammalian cell cultures (Brostrom and Brostrom, 2003), suggesting novel regulation of protein synthesis in neuropeptidergic cells. Reduced protein synthesis observed in mammalian cells treated with vasopression, angiotensin II and cholecystokinin (Brostrom et al., 1986; Kimball and Jefferson, 1990), agents that mobilize IP_3_R-mediated ER Ca^2+^ stores, can be rescued by addition of extracellular 2 mM Ca^2+^ during stimulation (Brostrom et al., 1986; Kimball and Jefferson, 1990; Sans et al., 2002). This suggests that extracellular Ca^2+^ entry counteracts ER-store Ca^2+^ effects on protein synthesis. Interestingly, when IP_3_R function is compromised in neurons, extracellular Ca^2+^ entry via SOCE is diminished (Venkiteswaran and Hasan, 2009). Thus, it is possible that a signaling cascade connects SOCE to protein translation, via IP_3_R.

Unlike NE cells, the rate of protein translation in *itpr^ku^* neurons was found to be no different from control neurons (Fig. S4A,B), suggesting that IP_3_R compensation of InR signaling is cell specific. Consistent with this, InR overexpression in cholinergic neurons of *itpr^ku^* did not rescue its viability on sucrose (Fig. S4C).

Peptide release from a subset of NE cells is regulated by IP_3_R-mediated Ca^2+^ transients from a subset of glutamatergic neurons (Jayakumar et al., 2016). Our results show that IP_3_R-mediated Ca^2+^ release also regulates protein translation in NE cells. Together, these observations illustrate the plurality of cellular processes controlled by IP_3_/Ca^2+^ signaling in the context of nutrient stress.

In summary, IP_3_R-mediated Ca^2+^ signaling helps maintain normal protein translation levels in NE cells, and this activity promotes systemic protein metabolism during larval development. On a nutrient-rich diet, loss of IP_3_R signaling is not detrimental, because dietary cues maintain insulin/TOR signaling, and thereby keep protein levels normal for completing development. Under starvation, dietary cues are lost. IP_3_/Ca^2+^ signaling possibly provides a nutrient-independent mechanism in order to maintain protein synthesis in cells essential to surviving nutrient stress, such as NE cells in which increased levels of cell surface receptors or neuropeptides might be required for modulating relevant neural circuits. As yet, there are no receptors or neuropeptides reported to be upregulated upon starvation in *dimm^+^* NE cells, but there is precedence to suggest that they might exist. For example, in starved *Drosophila*, the receptor for short Neuropeptide F is upregulated in the antenna (Root et al., 2011), and in starved mammals levels of agouti-related peptide, which affects appetite and feeding, are increased (Henry et al., 2015). A recent screen identified IP_3_/Ca^2+^-coupled neuropeptide receptors, on glutamatergic neurons, that are required for larval adaptation to nutrient stress (Jayakumar et al., 2016). Neuropeptides from NE cells that couple to such receptors might function during starvation in our model (Fig. S5).

By focusing on animal development, this study integrates cellular observations and organismal phenotype. Therefore, it sets the framework for the discovery of mechanistic details of how stimulus-coupled increases in cytosolic Ca^2+^ can regulate protein synthesis in a cell-specific manner, and how that consequently regulates protein metabolism in the whole animal.

## MATERIALS AND METHODS

### Fly husbandry and stocks

Flies were reared on ‘normal’ laboratory food (1 L recipe: 80 g corn flour, 20 g glucose, 40 g sugar, 15 g yeast extract, 4 ml propionic acid, 5 ml *p*-hydroxybenzoic acid methyl ester in ethanol, 5 ml ortho butyric acid) in an incubator at 25°C under 12 h/12 h light/dark conditions. Fly strains are listed in supplementary Materials and Methods.

### Larval nutritional stress assay

Eggs were collected on normal laboratory food for 6-8 h (depending on cross fecundity; ∼100 eggs per bottle) and allowed to mature for 88 h. For each genotype, six batches of 25 third instar larvae of similar size were transferred to a fresh vial of normal food or 100 mM sucrose in 1% agar. Pupae were scored 10 days after transfer. For development time, pupariation was scored every 24 h.

### Pupal volume measurement

Pupal volume was approximated by measuring the width and height from pupal pictures, and applying the volumetric formula for cylinders, πr^2^h.

### Weight, protein and TAG measurements on whole larvae

Thirty larvae were weighed on a microbalance (Shimadzu, Libror AEG 220). From this, ten larvae were homogenized in 1 ml of 0.2% Tween 20, followed by heating at 70°C for 10 min. Lysates were spun for 4 min at 4000 rpm (1500 ***g***). For the BCA assay, 5 µl of the supernatant was withdrawn and protein estimated following the manufacturer's protocol (Pierce BCA Protein Assay Kit). To 50 µl of supernatant, 150 µl of enzyme-substrate mix (BeneSphera GPS TAG Kit, Avantor Performance Materials) was added to measure TAG levels.

### RT-PCR

RNA was isolated from 10-12 larval brains at specified time points. cDNA synthesis was carried out as described (Pathak et al., 2015). All mRNA levels are reported as fold change normalized to *rp49* (*RpL32* – FlyBase). Primers are listed in supplementary Materials and Methods.

### *In vivo* protein translation assay

Neuronal cultures from late third instar larval brains were prepared as described (Deb et al., 2016). After 16-18 h, cultures were processed for *in vivo* protein synthesis labeling using the manufacturer's protocol provided with the Click-iT Plus OPP Protein Synthesis Assay Kit (C10458). Confocal fluorescence images were collected using an Olympus FV1000 at 60× with 0.5 µm *z*-stacks. Between 10 and 15 cells were imaged per dish and at least three independent dishes were cultured for each genotype. Identical confocal settings were used for all imaging. Total fluorescence in each channel for the entire stack was measured using ImageJ and the background in each channel for each individual cell was subtracted for the measured region of interest.
